# Long Non-Coding RNA LINC01929 Accelerates Progression of Oral Squamous Cell Carcinoma by Targeting the miR-137-3p/FOXC1 Axis

**DOI:** 10.3389/fonc.2021.657876

**Published:** 2021-04-21

**Authors:** Hongze Che, Yanhai Che, Zhimin Zhang, Qing Lu

**Affiliations:** ^1^Department of Endodontics, Hospital of Stomatology, Jilin University, Changchun, China; ^2^Department of Science and Education, Hospital of Stomatology, Jilin University, Changchun, China; ^3^Department of General Dentistry, Hospital of Stomatology, Jilin University, Changchun, China

**Keywords:** FOXC1, oral squamous cell carcinoma, LINC01929, ceRNA, miR-137-3p

## Abstract

Recently, additional long noncoding RNAs (lncRNAs) have been identified and their possible roles were investigated in a variety of human tumors. One of these lncRNAs, LINC01929, promoted the progression of some cancers, whereas its expression and biological function in human oral squamous cell carcinoma (OSCC) remains still mostly uncertain. The LINC01929 expression in OSCC tissues or cell lines was identified *via* quantitative real-time polymerase chain reaction. The cell counting kit-8, transwell migration, wound-healing, and flow cytometry assays were utilized to characterize the functions of LINC01929 in OSCC cells. The interactive relationships between LINC01929 and miR-137-3p, miR-137-3p and Forkhead box C1 (FOXC1) were investigated by the dual-luciferase activity assay. Our findings demonstrated that LINC01929 was highly expressed in OSCC tissue samples and cell lines, whereas miR-137-3p expression was downregulated. LINC01929 acted as a carcinogenic lncRNA with accelerated OSCC cell proliferation, migration and invasion, and suppression of apoptosis. We further indicated that LINC01929 facilitated tumor growth in xenograft mouse models. Mechanistically, LINC01929 acted as a sponge for miR-137-3p to elevate FOXC1 expression, which is the target of miR-137-3p. In addition, downregulated miR-137-3p expression rescued the suppressive behaviors of LINC01929 knockdown on the biological behaviors of OSCC cells. Taken together, LINC01929 functioned as a tumor-promoting lncRNA *via* the miR-137-3p/FOXC1 axis in OSCC, suggesting novel targets for OSCC therapy.

## Introduction

Oral squamous cell carcinoma (OSCC) accounts for a remarkable cases of human malignant tumors that occur in the head and neck region. It has the sixth highest incidence rate and a high mortality rate (1, 2). There have been remarkable advances in recent years in OSCC treatment, including surgery, radiotherapy, and chemotherapy. However, OSCC is accompanied with a low five-year survival rate (3, 4). The literature indicates that the progression of OSCC is accompanied with coding and non-coding gene disorders (5).

Scholars have defined long noncoding RNAs (lncRNAs) as non-coding RNAs that cannot encode genes and are less than 200 nucleotides in length (6–8). Numerous reports have suggested substantial role of lncRNAs in controlling gene expression at the transcriptional, post-transcriptional, and chromosomal levels, and also participated in the biological progression of proliferation, invasion, and apoptosis of tumor cells (9–11). Increasingly, additional lncRNAs have been shown to regulate mRNA expression at the post-transcriptional level by sponging miRNA with competitive endogenous RNA (ceRNA) mechanisms (12–15). Several researches pointed out that lncRNAs were involved in the development of OSCC (16–18). LncRNA BLACAT1 knockdown could inhibit invasion and migration of OSCC cells by sponging miR-142-5p (19). LncRNA ANRIL advanced the proliferation of OSCC cells and inhibited apoptosis by regulating the TGF-/Smad signaling pathway (20).

LINC01929 could promote tumor progression as a carcinogenic lncRNA in hepatocellular carcinoma and non-small cell lung carcinoma (21, 22). However, the role of LINC01929 in OSCC remains unclear.

We, in the present research, measured LINC01929 expression in OSCC tissues and OSCC cells and characterized its function *via in vitro* and *in vivo* experiments. We also attempted to evaluate the molecular mechanism by which LINC01929 plays a role in OSCC.

## Materials and Methods

### Tissue Collection

OSCC tissues and paired adjacent tissues of 27 confirmed patients were collected from the Stomatological Hospital of Jilin University. Prior to surgical removal, none of the patients received anticancer treatment. All patients signed informed consent. The confirmation of the present research could be achieved from the Ethics Committee of Stomatological Hospital of Jilin University.

### Cell Cultivation

Human oral keratinocytes (HOK) and OSCC cells (CAL-27, CAL-33, SCC-9, SCC-25, and HSC-3) were acquired from the Chinese Academy of Sciences. These were cultivated with DMEM with 10% FBS and maintained at 37°C in a humidified 5% CO_2_ incubator.

### Cell Transfection

ShRNA against LINC01929 (sh-LINC01929), control shRNA (sh-NC), pcDNA3.1-LINC01929 (pLINC01929), control pcDNA3.1 (pcDNA3.1), as well as pcDNA3.1-FOXC1 (pcDNA-FOXC1), and the empty control vector (pcDNA-NC) were utilized (GenePharma, Shanghai, China). To silence miR-137-3p, miR-137-3p inhibitor (miR-137-3p in) and negative control (miR-NC) were provided by GenePharma. Lipofectamine 2000 was applied to transfect the constructs separately into CAL-27 or SCC-9 cells.

### Quantitative Real-Time Polymerase Chain Reaction (qRT-PCR)

Total RNA of OSCC tissues and OSCC cells was extracted by TRIZOL reagent. Reverse transcription into cDNA was accomplished with the PrimerScript RT Reagent Kit. The reaction was performed on the ABI7500 system with SYBR Premix Ex Taq. Primers used in experiments are summarized in **Table 1**. GAPDH and U6 were used as internal controls for the cytoplasm and nucleus, respectively.

**Table 1:** The primer of referred genes.

Primers		Sequence (5’→3’)
LINC01929	Forward	5’-TGCCTTTGGTGTGGTCCTGTTTC -3’
	Reverse	5’-AAGATGCCCATACCAGACCTCCAG-3’
FOXC1	Forward	5’-GGCGAGCAGAGCTACTACC-3’
	Reverse	5’-TGCGAGTACACGCTCATGG-3’
GAPDH	Forward	5’-GGTCTCCTCTGACTTCAACA -3’
	Reverse	5’-GTGAGGGTCTCTCTCTTCCT-3’
miR-137-3p	Forward	5’ -CCATTCATTCGTTATTGCTTAAGA -3’
	Reverse	5’- TATGCTTGTTCTCGTCTCTGTGTC-3’
U6	Forward	5’- CTCGCTTCGGCAGCACA -3’
	Reverse	5’- AACGCTTCACGAATTTGCGT -3’

### Nucleus-Cytoplasm Separation

PARIS™ Kit was used to detect the cytoplasmic and nuclear distribution of LINC01929 in CAL-27 and SCC-9 cells. The expression levels of LINC01929, GAPDH, and U6 in cytoplasm and nucleus were detected by qRT-PCR.

### Cell Counting Kit-8 (CCK-8)

CAL-27 and SCC-9 cells (5x10^3^ cells/well) were used to coat onto 96-well plates for 24, 48, 72, and 96 h. Afterwards, CCK-8 reagent was mixed with the cells for 1 h incubation at 37 °C. Finally, the absorption value at 450 nm was measured with a microporous plate.

### Wound-Healing Assay

Transfected cells were coated onto 6-well plates and allowed to multiply until 100% confluence was reached. A scratch was then created with a 200 μL pipette tip, and the scratch distance was recorded through a microscope (Olympus) after 0 and 48 h in serum-free medium.

### Transwell Assay

The transwell assay was employed to measure invasion with Transwell chambers. Transfected cells in serum-free medium were added to the upper compartment, followed by adding DMEM with 10% FBS to the lower compartment. Once 24 h was passed after cultivation, the upper layer cells were gently removed, and the invaded cells in the lower layer were immobilized by 4% paraformaldehyde and crystal violet. Five fields were randomly selected and photographed with a microscope.

### Flow Cytometry Assay

The annexin V-fluorescein isothiocyanate (FITC)-apoptosis detection kit was used. Concisely, we attempted to wash transfected cells with PBS for two times, followed by staining with Annexin V-FitC in darkness at room temperature for 5 min and then with 10 μL of PI for 15 min. Finally, flow cytometry was employed for detecting apoptosis.

### Luciferase Reporter Assay

Mutant plasmids (FOXC1-MUT, LINC01929-MUT) and wild-type plasmids (FOXC1-WT, LINC01929-WT) were constructed using the pmirGLO double-luciferase vector. Once we co-transfected the plasmids with miR-137-3p mimics or NC mimics, Renilla luciferase activity was detected.

### Tumor Xenograft Assay

Immunodeficient female BALB/c nude mice (4‐6 weeks old) were provided by Shanghai Laboratory Animal Company and maintained at the Animal Laboratory Center of Jilin University. The animal experiment ethics committee of Jilin University approved the animal experiments. The knockdown transfected cells (5 × 10^6^/mL, 100 μL) were then injected subcutaneously into nude mice (n = 3/group). We attempted to quantify tumor volumes once a week. The mice were killed four weeks after the mold was made. The OSCC tissues of mice were removed, weighed, photographed, and stored for subsequent qRT-PCR and Western blotting.

### Western Blotting

According to the kit instructions, radioimmunoprecipitation assay (RIPA) protein lysis buffer was applied to extract protein lysates from OSCC tissues or OSCC cells, and 10% SDS-PAGE was applied for separation, followed by transferring the protein to PVDF membrane, and blocking with skim milk. The blot was incubated at 4°C with primary and secondary antibodies, separately. The band signal was visualized through BeyoECL.

### Statistical Analysis

Data analyses were undertaken with GraphPad PRISM 6 and SPSS 17.0 software. The findings were summarized as mean ± standard deviation (SD), from three independent experiments and analyzed *via* the Student’s *t*-test. *P* < 0.05 was served to show statistical significance.

## Results

### LINC01929 Was Highly Expressed in OSCC Tissues and OSCC Cells

**Figure 1:**
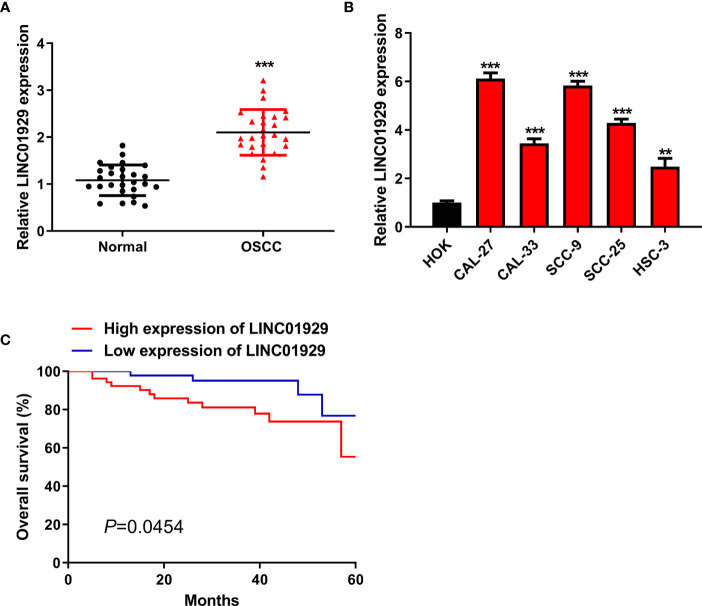
High LINC01929 expression in OSCC tissues and OSCC cells. **(A)** LINC01929 expression was measured by qRT-PCR in OSCC tissues and paired non-tumor tissues (n = 27). **(B)** The LINC01929 expression was identified by qRT-PCR in HOKs and OSCC cells. **(C)** Survival curves were generated with Kaplan-Meier to measure the correlation between LINC01929 expression and overall survival of OSCC patients. **p < 0.01, ***p < 0.001.

In our study, LINC01929 expression was detected for the first time in 27 OSCC tissues. The findings unveiled that LINC01929 expression was remarkably elevated in OSCC tissues (**Figure 1A**). To explore the markedly upregulated LINC01929 expression in OSCC, we attempted to divide patients into groups of high LINC01929 expression and low LINC01929 expression. This revealed that elevated LINC01929 expression was linked with advanced lymph node metastasis (**Table 2**). We also measured the LINC01929 expression in HOKs and OSCC cells, including CAL-27, CAL-33, SCC-9, SCC-25, and HSC-3. LINC01929 expression was upregulated in OSCC cells, while CAL-27 and SCC-9 cells showed the highest expression (**Figure 1B**). Thus, CAL-27 and SCC-9 cells were selected for subsequent experiments. Kaplan-Meier assays suggested that patients with higher LINC01929 expression had poorer overall survival rates than those with lower LINC01929 expression (**Figure 1C**). In general, the level of LINC01929 was higher in OSCC tissues and OSCC cells and correlated with poorer OSCC clinical outcomes.

**Table 2:** Correlation between LINC01929 expression and clinicopathological factors in patients of OSCC.

Factors	LINC01929 expression		*P*-value
	Low	High	
Age (years)			0.4743
<60	5	8	
≥60	7	7	
Gender			0.6471
Male	4	6	
Female	8	9	
Tumor size (cm)			0.3928
<5	8	10	
≥5	4	5	
TNM stage			0.0885
I-II	6	7	
III-IV	6	8	
Lymph node metastasis			0.0183*
Yes	2	13	
No	10	2	

*P < 0.05 was considered statistically significant.

### LINC01929 Regulated Proliferation, Migration, Invasion, and Apoptosis of OSCC *In Vitro*

**Figure 2:**
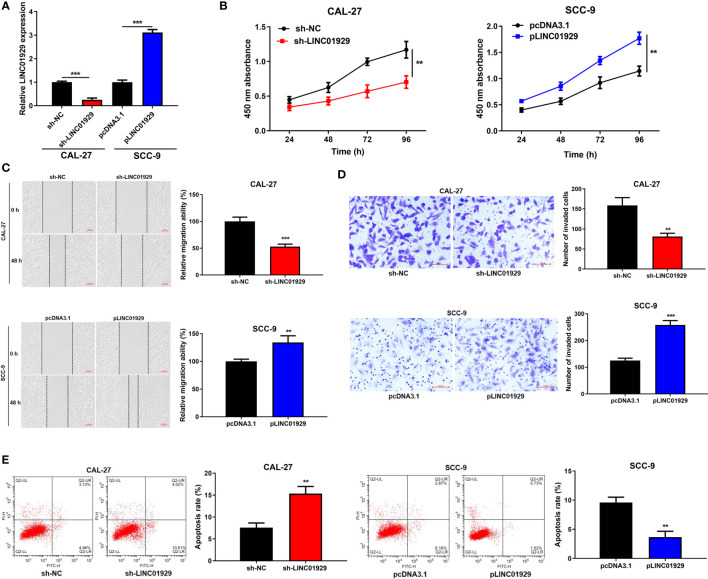
LINC01929 regulated proliferation, migration, invasion, and apoptosis of OSCC *in vitro*. **(A)** qRT-PCR analysis was used to examine the efficiency of sh-LINC01929 and pLINC01929. **(B)** Proliferation was monitored by CCK-8 assay after transfecting sh-LINC01929 or pLINC01929 into selected OSCC cells. **(C, D)** Wound-healing assay and transwell assay were employed to assess migration and invasion after LINC01929 knockdown or overexpression. **(E)** The effects of sh-LINC01929 or pLINC01929 on apoptosis was detected by flow cytometry. **p < 0.01, ***p < 0.001.

To detect the biological function of LINC01929 in OSCC cells, we firstly used sh-LINC01929 to knockdown LINC01929 in CAL-27 cells, while pLINC01929 to overexpress LINC01929 in SCC-9 cells. qRT-PCR highlighted the successfully transfection of sh-LINC01929 and pLINC01929 (**Figure 2A**). CCK-8 assay indicated that downregulation of LINC01929 dramatically reduced, whereas LINC01929 overexpression promoted proliferation (**Figure 2B**).

As illustrated in **Figures 2C, D**, migration and invasion in the sh-LINC01929 group were limited and the pLINC01929 group were increased compared with the control group. Then, flow cytometry was used to examine apoptosis, and the data showed that apoptosis of OSCC cells was notably elevated after LINC01929 knockdown, while inhibited after LINC01929 upregulation (**Figure 2E**). In summary, the above-mentioned findings indicated that LINC01929 regulated cell proliferation, migration, invasion, and apoptosis of OSCC cells.

### LINC01929 Sponged miR-137-3p in OSCC Cells

**Figure 3:**
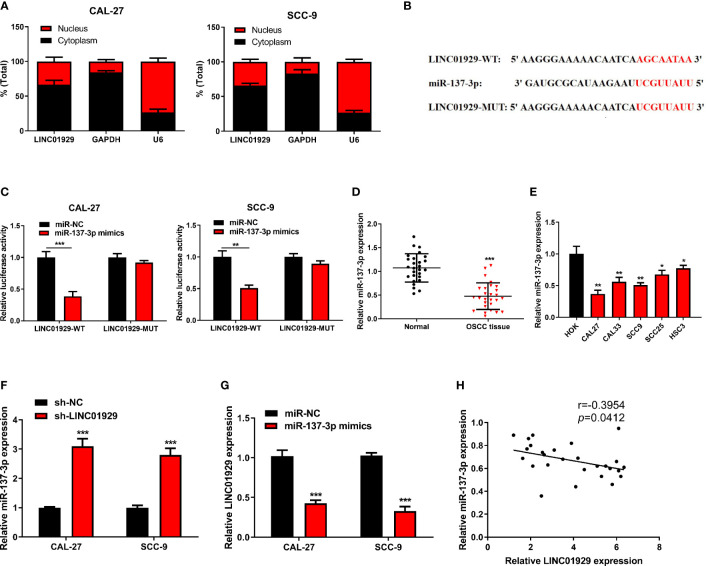
LINC01929 sponged miR-137-3p in OSCC cells. **(A)** Identification of LINC01929 expression by nuclear and cytoplasmic fractionation to analyze its subcellular location. **(B)** The binding site between LINC01929-MUT and miR-137-3p was predicted with bioinformatics analysis. **(C)** The luciferase gene reporter assay was applied to test the binding relationship between LINC01929 and miR-137-3p mimics. **(D, E)** The miR-137-3p expression was detected by qRT-PCR in OSCC tissues and OSCC cells. **(F)** The miR-137-3p expression was identified in LINC01929 knockdown OSCC cells. **(G)** The LINC01929 expression was tested after transfecting with miR-137-3p mimics into OSCC cells. **(H)** Spearman’s correlation analysis was used to analyze the relevance between LINC01929 and miR-137-3p. *p < 0.05, **p < 0.01, ***p < 0.001.

The subcellular distribution of lncRNAs determines their biological actions. Through the nuclear-cytoplasmic fractionation assay, LINC01929 was mainly found in the cytoplasm (**Figure 3A**). Therefore, we speculated that LINC01929 regulated the function of OSCC through a ceRNA mechanism. The miRDB prediction revealed a sequence that suggested that binding of miR-137-3p to LINC01929 was possible (**Figure 3B**). As depicted in **Figure 3C**, dual-luciferase activity assay verified that miR-137-3p mimics downregulated the luciferase activity of LINC01929-WT. However, the effect of miR-137-3p expression on LINC01929-MUT was not noticeable. Through qRT-PCR, we discovered that miR-137-3p expression was attenuated in OSCC tissues compared with paracancer tissues (**Figure 3D**), and was downregulated in OSCC cells (**Figure 3E**). In addition, after LINC01929 expression was knocked down in OSCC cells, miR-137-3p expression was markedly elevated (**Figure 3F**). When miR-137-3p was upregulated, this was associated with a remarkable inhibition in LINC01929 expression (**Figure 3G**). **Figure 3H** depicts that the lower miR-137-3p expression in OSCC tissues was negatively correlated with that of LINC01929. In conclusion, LINC01929 could target miR-137-3p in OSCC cells.

**Figure 4:**
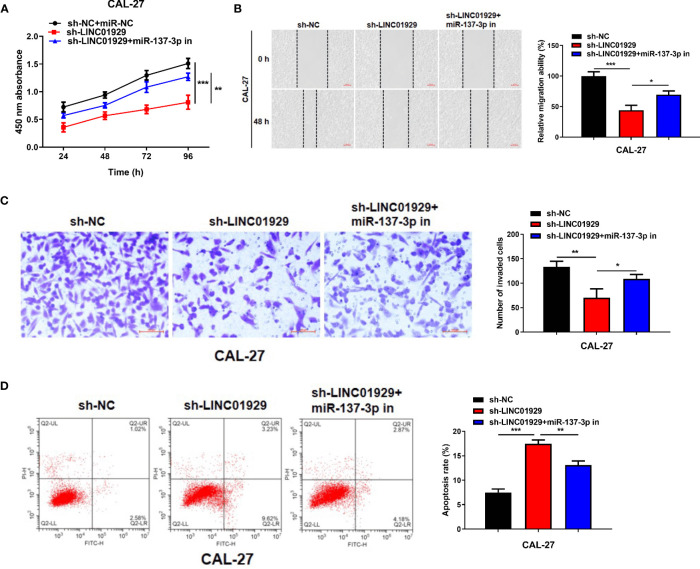
MiR-137-3p inhibitor restored the effect of LINC01929 knockdown on OSCC cells. **(A)** CCK-8 assay showed CAL-27 cells proliferation after transfection of sh-NC, sh-LINC01929 and sh-LINC01929+miR-137-3p inhibitor. **(B, C)** Wound healing assay and transwell assay showed CAL-27 cells migration and invasion after transfection of sh-NC, sh-LINC01929 and sh-LINC01929+miR-137-3p inhibitor. **(D)** The flow cytometry was applied to measure apoptosis in each group.

We conducted *in vitro* experiments to explore whether the binding between miR-137-3p and LINC01929 affects OSCC progression. Sh-LINC01929 and miR-137-3p inhibitor were co-transfected into CAL-27 cells. The cell proliferation of sh-LINC01929 cells was rescued by the introduction of miR-137-3p inhibitor (**Figure 4A**). Wound healing and transwell assays demonstrated that miR-137-3p inhibitor significantly reversed sh-LINC01929-attenuated migration and invasion activity of CAL-27 cells (**Figure 4B, C**). Furthermore, flow cytometry assay indicated co-introduction of miR-137-3p remarkedly decreased cell apoptosis compared with sh-LINC01929 group (**Figure 4D**).

### MiR-137-3p Directly Interacted With FOXC1

**Figure 5:**
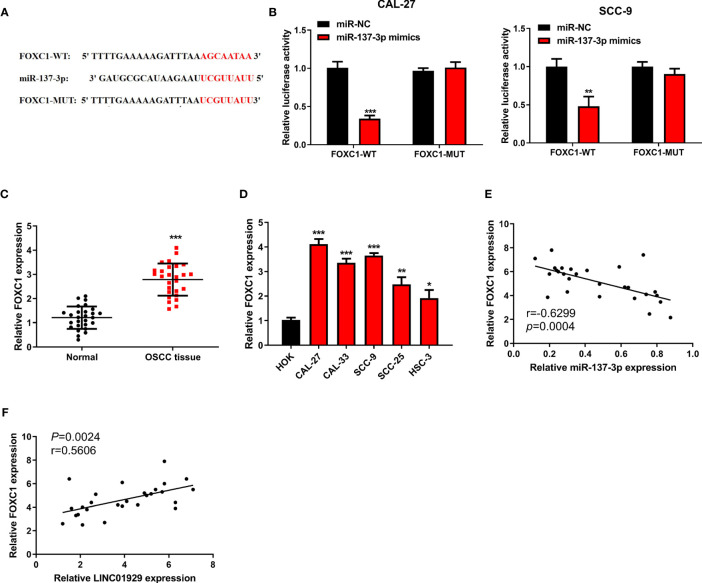
MiR-137-3p directly interacted with FOXC1. **(A)** Potential binding and mutant sequences were constructed. **(B)** The putative site of miR-137-3p in the FOXC1 3’UTR was detected by the dual-luciferase reporter assay. **(C)** FOXC1 expression was monitored by qRT-PCR in OSCC tissues and paired adjacent normal tissues. **(D)** The FOXC1 expression was measured by qRT-PCR in OSCC cells and the HOKs. **(E)** Correlation analysis of miR-137-3p expression with FOXC1 expression. **(F)** Correlation analysis of LINC01929 expression with FOXC1 expression.

We sought to identify the target of miR-137-3p and found that FOXC1 contained a putative binding site for miR-137-3p (**Figure 5A**). We found that luciferase activity of cells transfected with FOXC1-WT was notably inhibited by miR-137-3p mimics (**Figure 5B**). Moreover, we found that FOXC1 expression was notably upregulated in OSCC tissues compared with neighbor non-tumor tissues (**Figure 5C**). Furthermore, FOXC1 was upregulated in OSCC cells (**Figure 5D**). Finally, outcomes of Spearman’s correlation analysis suggested that FOXC1 was negatively correlated with miR-137-3p, and LINC01929 expression and FOXC1 expression showed a notable positive correlation (**Figures 5E, F**). The above-mentioned findings highlighted that LINC01929 sponged miR-137-3p, acted as a ceRNA, and released FOXC1 from the inhibitory effect of miR-137-3p.

### The LINC01929/miR-137-3p/FOXC1 Axis Regulated the Behavior of OSCC Cells

**Figure 6:**
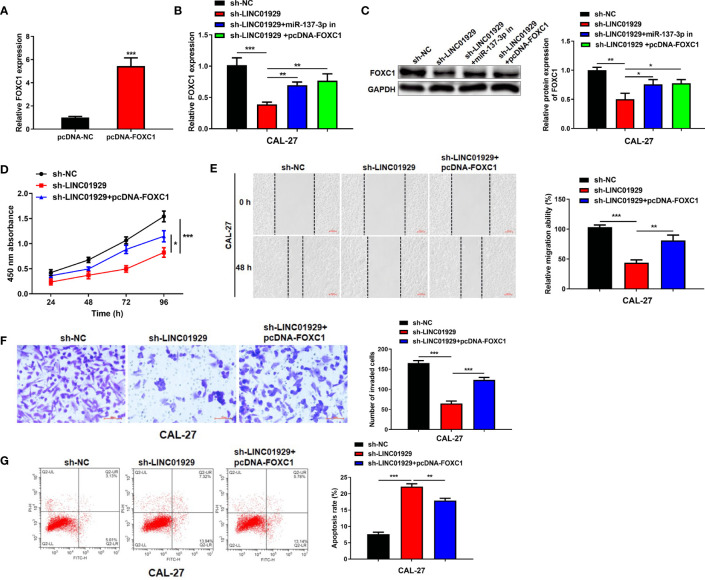
The LINC01929/miR-137-3p/FOXC1 axis regulated the behavior of OSCC cells. **(A)** The efficiency of FOXC1 overexpression was measured by qRT-PCR. **(B)**The mRNA and **(C)** protein expressions of FOXC1 were identified by qRT-PCR and Western blotting in OSCC cells transfected with sh-NC, sh-LINC01929, sh-LINC01929+miR-137-3p in and sh-LINC01929+pcDNA-FOXC1. **(D)** The CCK-8 assay was applied to identify the proliferation in each group. The migration **(E)** and invasion **(F)** in each group were identified by wound-healing assay and transwell assay. **(G)** The flow cytometry was applied to measure apoptosis in each group.

We speculated that LINC01929/miR-137-3p may influence OSCC progression *via* FOXC1. The findings highlighted that transfection of pcDNA-FOXC1 remarkably elevated its expression level (**Figure 6A**). qRT-PCR and Western blotting indicated that miR-137-3p expression downregulation or FOXC1 expression upregulation could reverse the LINC01929 knockdown-mediated inhibition on FOXC1 expression (**Figures 6B, C**). CCK-8 assay revealed that FOXC1 overexpression could restore the LINC01929 knockdown-mediated inhibition on proliferation (**Figure 6D**). Furthermore, FOXC1 overexpression had a partial reversal effect on the migration and invasion changes in CAL-27 cells caused by LINC01929 knockdown (**Figures 6E, F**). Flow cytometry assay confirmed that the enhanced apoptosis of OSCC cells caused by sh-LINC01929 could be reversed by FOXC1 upregulation (**Figure 6G**). In general, LINC01929 promoted proliferation, migration, and invasion of OSCC cells *via* the miR-137-3p/FOXC1 axis.

### Downregulation of LINC01929 Inhibited Tumor Growth *In Vivo*

**Figure 7:**
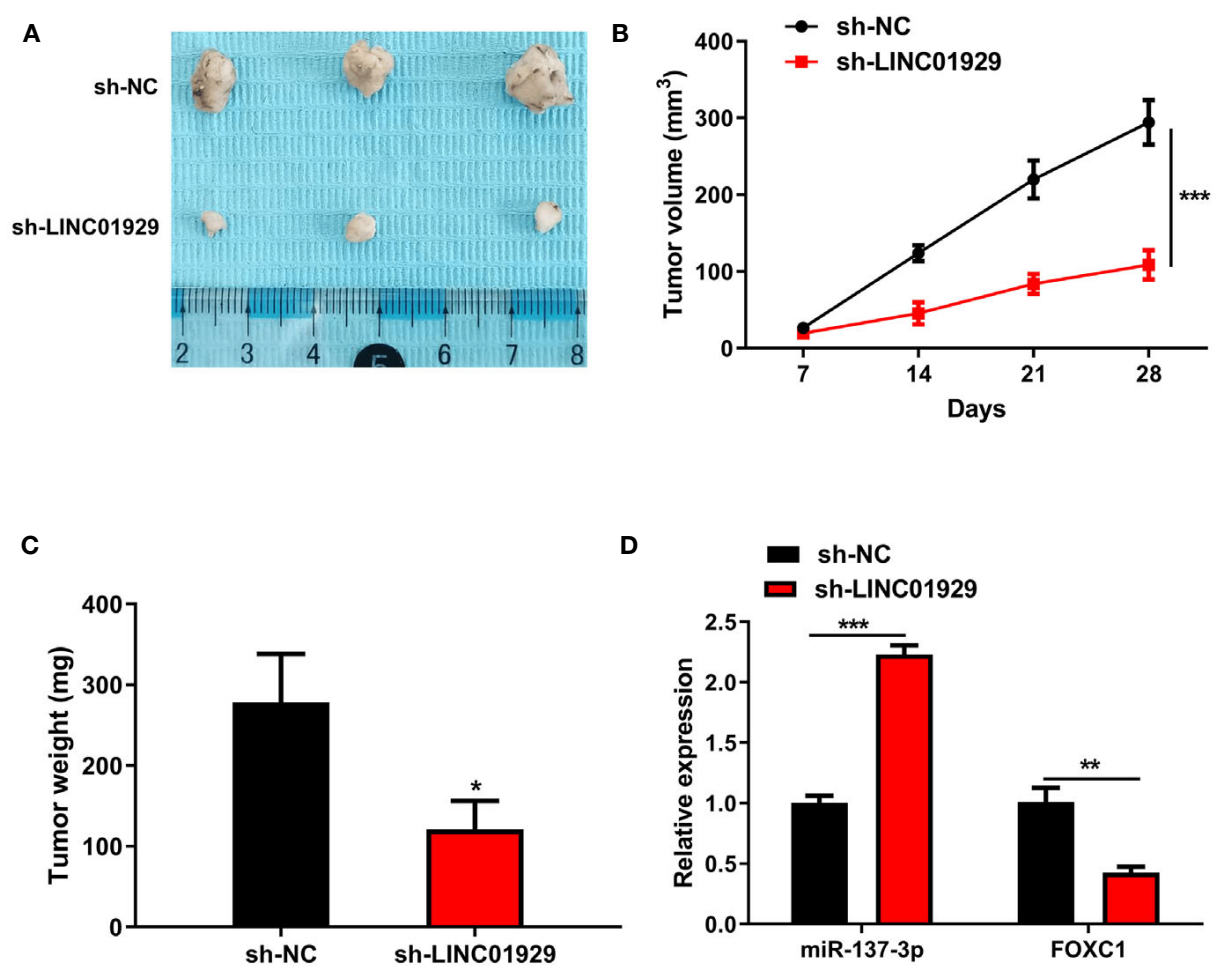
Downregulation of LINC01929 inhibited tumor growth *in vivo*. **(A)** Tumor images at 28 days post-xenotransplantation from the sh-LINC01929 groups and sh-NC groups. **(B)** Quantification of tumor volume was undertaken every seven days after the xenotransplantation. **(C)** Tumor weight was monitored after the experiment. **(D)** qRT-PCR was employed to identify miR-137-3p expression and FOXC1 expression in xenograft tumors. *p < 0.05, **p < 0.01, ***p < 0.001.

Immuno-deficient nude mice were used in a xenograft model to detect the influence of LINC01929 on OSCC progression. LINC01929 knockdown markedly attenuated tumor growth as well as tumor weight (**Figures 7A–C**). qRT-PCR assay confirmed enhanced miR-137-3p and reduced FOXC1 expression in sh-LINC01929 tumors (**Figure 7D**). The above-mentioned consequences demonstrated that downregulation of LINC01929 expression noticeably limited tumor growth *in vivo*.

## Discussion

Several recently conducted researches have pointed out that lncRNA expression disorders actively participate in the development of tumors (23–27). In OSCC, more and more lncRNAs have been shown to be involved in changing the biological functions of tumor cells (28–30). For instance, Shao et al. stated that LncRNA AC007271.3 accelerates proliferation, invasion, and migration of OSCC cells, and suppresses apoptosis of OSCC through the Wnt/β-catenin signaling pathway (31). Xu et al. pointed out that lncRNA FezF1-AS1 accelerates the occurrence and development of OSCC by directly binding to miR-196 (32). Scholars pointed out that LINC01929 has carcinogenic effects in several types of cancer (21, 22). In the present research, we sought to understand the functions and molecular mechanisms of LINC01929 in OSCC. We revealed that LINC01929 was highly expressed in OSCC tissues and OSCC cells. Functional experiments indicated that LINC01929 promoted proliferation, migration, and invasion of OSCC cells *in vitro*, as well as regulated tumor growth *in vivo*. This demonstrates that LINC01929 also has an oncogenic role in OSCC.

The function of lncRNA depends on its downstream binding molecules, such as ceRNA, which can competitively bind miRNA and thus regulate the changes of targeted mRNA expression. In gastric cancer, lncRNA MT1JP as ceRNA sponges miR-92a-3p to regulate the expression of FBXW7 and then influence the progression of gastric cancer (33). LINC01087 is enhanced in breast cancer, and LINC01087 affects the expression of ROCK1 by sponging miR-335-5p, thus affecting the migration and invasion of breast cells (34). Using bioinformatics and dual-luciferase activity assays, we verified that LINC01929 and miR-137-3p could fully bind to each other. The miR-137-3p expression was downregulated in OSCC tissues and OSCC cells compared with normal materials. Some reports suggest that miR-137-3p acts as a tumor suppressor gene in cancer. In colorectal cancer, miR-137-3p is low expressed and suppresses migration of colorectal cancer cells *via* regulating a KDM1A-dependent EMT process (35). Overexpression of miR-137-3p inhibits the tumor growth of prostate cancer by regulating the JNK3/EZH2 signal pathway (36). In the present research, suppressing miR-137-3p expression partially abolished the inhibition of proliferation, migration, and invasion of OSCC cells upon LINC01929 knockdown. Overall, the outcomes highlight that LINC01929 knockdown suppresses the growth of OSCC cells by regulating miR-137-3p expression.

FOXC1, which is also known as FREAC3, Fkh-1, or Mf1, is a single exon gene which encodes a 533 amino acid protein located at 6p25 in the nucleus, where it can bind to DNA and regulate gene expression (37–40). In recent years, FOXC1 has been found to play a key role in tumorigenesis. FOXC1 expression is upregulated in some types of cancer and participates in tumor formation as an oncogene. Knockdown of FOXC1 expression by siRNA dramatically inhibits proliferation, migration, and invasion of basal-like breast cancer cell lines (41). FOXC1 upregulates the expression levels of CCL2 and CXCR1 by directly binding to their promoters in hepatocellular carcinoma cells. The elevated expression of CXCR1 accelerates invasion and metastasis of hepatocellular carcinoma cells (42). FOXC1 functions as an oncogene in OSCC cells. LncRNA-FOXCUT and FOXC1 are overexpressed in OSCC patients and knockdown of either FOXCUT or FOXC1 inhibits the proliferation and migration of OSCC cell Tca8113 and SCC-9 (43). FOXC1-silenced OSCC cells exhibits decreased growth and migration, accompanied by a downregulation of MMP-2 and MMP-9 (44). Employing the dual-luciferase activity assay, we confirmed that FOXC1 is a direct target gene of miR-137-3p. It could be elevated in OSCC tissues and OSCC cells. It accelerated the proliferation, invasion, and migration of OSCC cells. FOXC1 partially reversed the changes in biological behaviors induced by sh-LINC01929.

**Figure 8:**
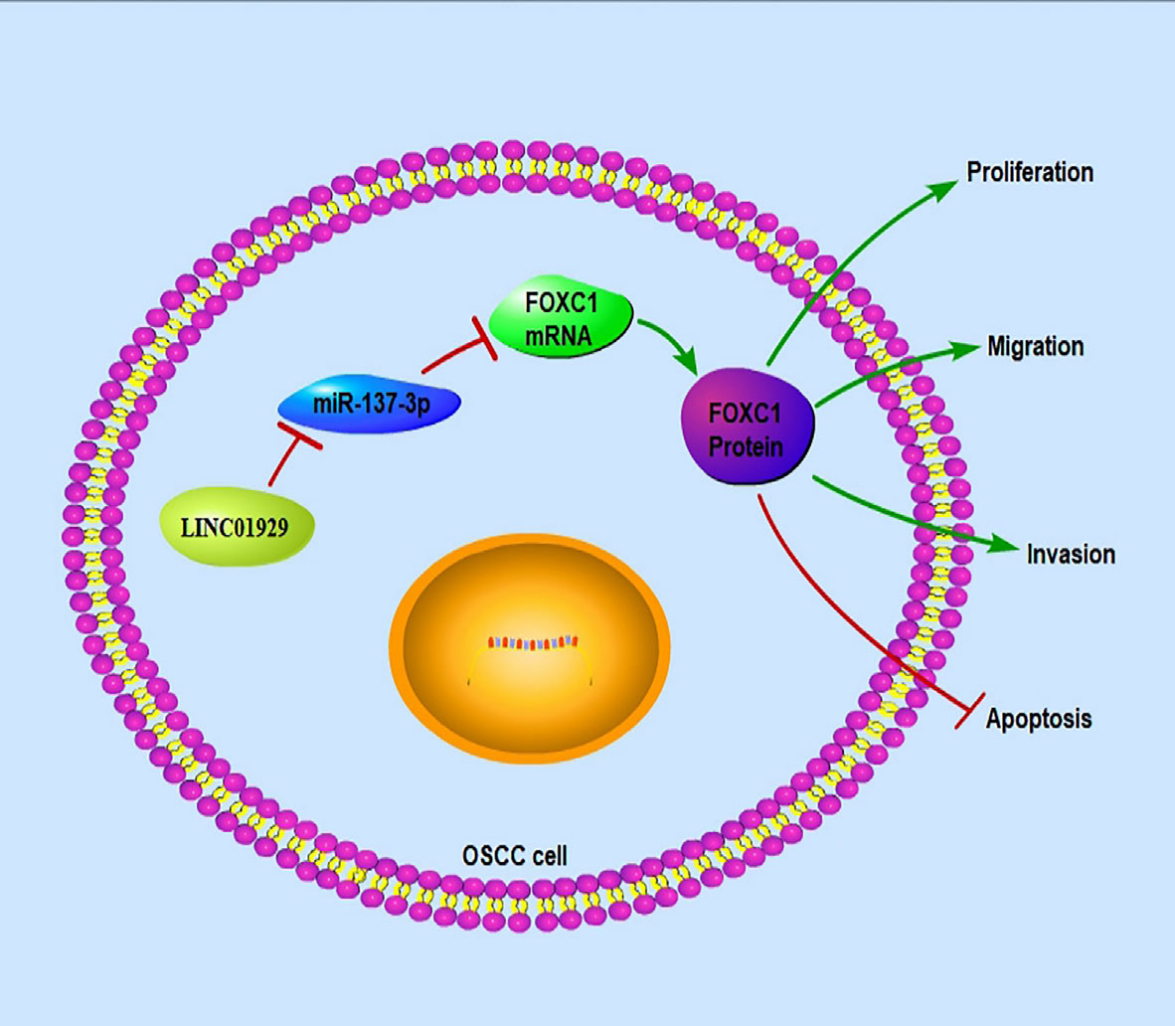
Schematic of the LINC01929/miR-137-3p/FOXC1 axis in OSCC cells.

In summary, the present research elucidated that LINC01929 targets FOXC1 *via* miR-137-3p to enhance the OSCC (**Figure 8**). As far as we know, this is the first research aimed to characterize the function and mechanism of the LINC01929 in OSCC. The outcomes may provide new targets for the diagnosis and treatment of OSCC.

## Data Availability Statement

The original contributions presented in the study are included in the article. Further inquiries can be directed to the corresponding authors.

## Ethics Statement

The studies involving human participants were reviewed and approved by Stomatological Hospital of Jilin University. The patients/participants provided their written informed consent to participate in this study. The animal study was reviewed and approved by The First Hospital of Jilin University.

## Author Contributions

HC and QL designed experiments and wrote the manuscript. HC and YC performed the experiments and data analysis. All authors critically discussed the results and the manuscript. ZZ and QL supervised the project and gave final approval. All authors contributed to the article and approved the submitted version.

## Funding

This work was supported by the China Postdoctoral Science Foundation funded project (2020M670865).

## Conflict of Interest

The authors declare that the research was conducted in the absence of any commercial or financial relationships that could be construed as a potential conflict of interest.
